# Effect of Moxibustion on Intestinal Microbiome in Acute Gastric Ulcer Rats

**DOI:** 10.1155/2019/6184205

**Published:** 2019-12-18

**Authors:** Qi-da He, Miao-sen Huang, Long-bin Zhang, Jia-cheng Shen, Lin-yu Lian, Yuan Zhang, Bao-hua Chen, Cai-chun Liu, Lin-chao Qian, Mi Liu, Zong-bao Yang

**Affiliations:** ^1^Cancer Research Center, School of Medicine, Xiamen University, Xiamen 361005, China; ^2^College of Acupuncture and Moxibustion, Fujian University of Traditional Chinese Medicine, Fuzhou 350122, China; ^3^College of Acupuncture and Moxibustion, Hunan University of Traditional Chinese Medicine, Changsha 410208, China

## Abstract

In Traditional Chinese Medicine (TCM), moxibustion had been used for thousands of years. Many clinical case reports and scientific studies had proved that moxibustion had a good effect in treating acute gastric ulcer (AGU). Some studies had shown that the relative content and species of bacteria in the intestinal would be changed when gastric mucosal injury happened. However, there was little research on the effect of intestinal microbiome with AGU rats that were treating by moxibustion. This study is aimed at analyzing the effect of fecal microbiome in rats with AGU by the 16S rDNA sequencing technology. Male SD rats were established by orally feeding once with 70% ethanol at 4 ml/kg except the control group, then treated by moxibustion in the stomach meridian group (“Liangmen,” “Zusanli”) and the gallbladder meridian group (“Riyue,” “Yanglingquan”) for 5 days. The 16S rDNA sequencing technology analysis of feces combined with histopathological methods and molecular biological detection methods was used to evaluate the therapeutic mechanism of moxibustion on AGU. AGU brought cause changes in the number and species of intestinal bacteria. Moxibustion on stomach meridian group could reduce the area of gastric mucosal injury and regulate the relative content of GAS and EGF. Moreover, moxibustion on the stomach meridian group could increase the relative content and species of beneficial bacteria in the intestine of rats with AGU. The relative abundance of intestinal probiotics was significantly upregulated in Alphaproteobacteria, Actinomycetales, and Bacillales. In addition, moxibustion might promote the repair of gastric mucosal injury by increasing the number and species of beneficial bacteria in the intestine.

## 1. Introduction

Acute gastric ulcer (AGU) is a common digestive tract disorder in clinics. There are about ten million patients with gastric ulcer in mainland China [1]. Gastric mucosal injury has been proved to be the initial cause of various refractory diseases [2]. AGU is treated by *Helicobacter pylori* eradication therapy, which is anti-inflammatory, regulating gastric acid secretion, in modern medicine [3]. But, these therapies make the disease prone to relapse and the long-term drug treatment is of high cost [4]. On the other hand, drug therapy often has side effects. Our previous studies have proved that the ethanol damage model could cause acute gastric mucosal damage quickly and efficiently. Therefore, we chose the ethanol modeling method to prepare an AGU model.

Moxibustion usage is safe, effective, and conveniently complementary in therapies [5, 6]. Many clinical reports and experimental studies had proved that moxibustion had a good therapeutic effect on gastric mucosal injury [7, 8]. Moxibustion is to stimulate specific points by the burning heat of moxa [9]. In traditional Chinese medicine, the stomach meridian had an important connection with the stomach. Stomach diseases can be treated by stimulating the acupoints on the stomach meridian [10, 11]. In our previous studies, we found that moxibustion could repair the pathological morphology of gastric mucosal injury, balance the brain gut peptides, and regulate the metabolites [10, 12]. Some studies had shown that gastric diseases could affect the intestinal microbiome [13, 14]. However, there are little studies about the effect of moxibustion on intestinal flora in rats with AGU.

In the study, the effects of moxibustion on the content and species of bacteria in the intestine of gastric ulcer rats were examined by 16s rDNA sequencing technology. In addition, the study evaluated the pathological morphology and molecular biology of gastric mucosa after moxibustion treatment.

## 2. Materials and Methods

### 2.1. Animals

The process of the experiment was approved by the Animal Ethics Committee of Xiamen University (no: SCXK160803004). All rats were raised in the animal room (24∼26°C) of Medical College of Xiamen university. The experimental procedures were in accordance with the requirements of the National Institutes of Health for the Care and Use of Laboratory Animals.

Twenty-four Sprague Dawley rats (160 ± 20 g weight) were bred for a week to adapt to the environment of animal housing. Subsequently, all rats were randomly divided into a control group, acute gastric ulcer (AGU) group, stomach meridian (ST) group, and gallbladder meridian (GB) group.

### 2.2. Establishment of Gastric Ulcer Rats

**Figure 1:**
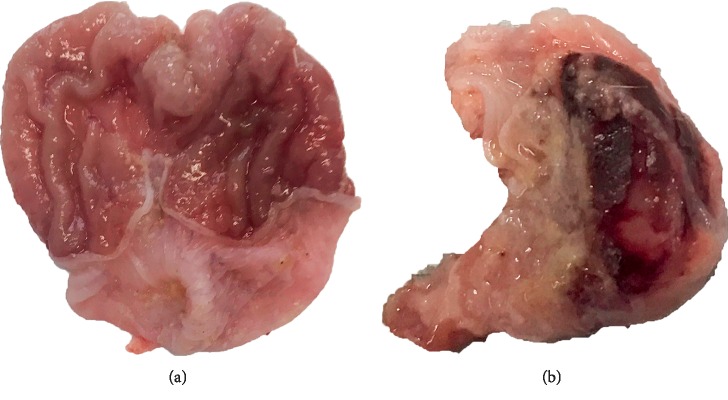
Comparison of gastric mucosal morphology between the (a) control group and (b) AGU group of rats.

According to the literature, the AGU model was established by intragastric administration of 70% ethanol with 4 ml/kg [15]. The AGU model was established successfully after 3 hours. All groups were established the AGU model except for the control group. After successful molding, the obvious ulcer focal can be seen, which indicates that the AGU model had been successfully prepared (Figure 1).

### 2.3. Moxibustion Treatment

Some studies had proved that the points of the stomach meridian had a good therapeutic effect on gastric mucosal injury [10, 16, 17]. Meanwhile, our previous studies proved that GU could be treated effectively by acupuncture at Liangmen (ST 21) and Zusanli (ST 36), which belong to the stomach meridian [18–21]. Therefore, “Liangmen” (ST21), “Zusanli” (ST36), “Riyue” (GB24), and “Yanglingquan” (GB34) were selected for treatment by moxibustion (Supplementary [Supplementary-material supplementary-material-1]). The location of acupoints in rats was according to “The Veterinary Acupuncture of China.” The moxa (Hanyi, Nanyang, China; high: 16 mm; diameter: 18 mm) was specially used for the rats in this experiment.

The day after successful modeling, the rest of the groups' rats were treated by moxibustion except the control group and AGU group for 5 days. All rats were sterilized at acupoints by 70% alcohol before treatment. Also, the moxa cone was placed on top of the selected spots, and moxa-burning sticks were fixed to ensure that lit ends were 2 cm away from the skin. The rats of the ST group and GB group were treated by moxibustion once daily for 15 min in a total of 5 days. The two points on the same side were selected for each treatment, and points in other side would be chosen at the next time of treatment.

### 2.4. Evaluation of Gastric Ulcer Index

All rats were anesthetized by inhaling of isoflurane after treatment. The stomach was cut along the greater curvature and cleaned with 0.9% Nacl solution. The area of gastric ulcer was measured by vernier caliper, and the index of gastric ulcer was calculated. The degree of gastric mucosal injury was scored according to the following categories: 0 = gastric mucosal integrity; 1 = the gastric mucosal had a small round ulceration; 2 = the ulceration <2 mm; 3 = the ulceration 2-3 mm; 4 = the ulceration 3-4 mm; and 5 = the ulceration >4 mm. The score was multiplied by 2 when the width of ulceration was <1 mm.

### 2.5. Histopathology

After evaluation of the gastric ulcer index, the gastric mucosa (0.5 cm × 0.5 cm) was collected and washed by sterile 0.9% NaCl solution on aseptic table, and the gastric ulcer index of the gastric ulcer rats was calculated. The samples were placed in 10% formalin solution and fixed for 48 hours, gradient ethanol dehydration was performed, and paraffin embedded. Then, the samples were sectioned with 5 *μ*m thickness. After hematoxylin and eosin staining, the pathological morphology of gastric mucosa was observed under an optical microscope. At the same time, an image acquisition system was used to collect pathological images of gastric mucosa of rats with different multiples.

### 2.6. Quantitative Real-Time PCR (qPCR)

Rats in each group were sacrificed by isoflurane after 5 days. The gastric mucosa (0.5 cm × 0.5 cm) of rats was collected for qPCR detection. Total RNA from gastric mucosa was extracted by the method of Trizol and reverse transcription into cDNA. Then, the amplification cycle was performed in PCR amplifier. CT values were derived after the amplification cycle, and the relative expression of target genes was analyzed by the method of 2^−ΔΔCT^.

### 2.7. Detection Methods and Date Analysis of Fecal Microbiome

The feces of all rats were collected under sterile environment before sacrifice. Then, the 16S rDNA sequencing and RFLP analysis were used to detect the fecal flora. The total DNA was extracted according to the kit (Sigma-Aldrich DNB200-50RXN, USA). The concentration and purity of DNA samples were detected. Then, a Nucleic Acid Quantizer Nano Drop ND-1000 was used to quantify the extraction efficiency of DNA by the ratio of A260/230 and A260/280. On the other hand, the target fragment of the 16S rDNA gene was amplified from the fecal flora solution: the DNA of *Escherichia coli* was used as a template, then the 16S rDNA of the bacteria was used as a universal primer for PCR amplification, and the microbial regions V3 and V4 were amplified. A DNA Sample Preparation Kit (Illumina TruseqTM, USA) was used to build databases based on mixed samples. Afterward, the DNA sequence was detected (Illumina Hiseq2500, USA). Next, the nonrepetitive sequences were clustered by OTU. The chimeras were removed to obtain the corresponding OTU sequences during the clustering process. The similarity of each OTU sequence was compared according to Sliva database (http://www.arb-silva.de). Data were statistically analyzed by PCA analysis and analysis of similarities (ANOSIM). Among them, the similarity analysis of Analysis of Similarties (ANOSIM) and the LEFSE analysis were used to analyze and mark species with differences in abundance between groups. Finally, the PCA analysis method was used to eliminate noise and redundant data which could reduce the dimension of the original complex data and sort the samples by the species abundance matrix.

## 3. Results

### 3.1. Gastric Ulcer Index Detection

The gastric ulcer index was observed to determine the healing of gastric injury in rats. Comparing with the AGU group, the index of gastric ulcer was significantly reduced in group ST. Moreover, the gastric ulcer index was also reduced in the GB group, but the GB group was not significantly different than the GU group (Table 1).

### 3.2. Histopathological Observation

**Figure 2:**
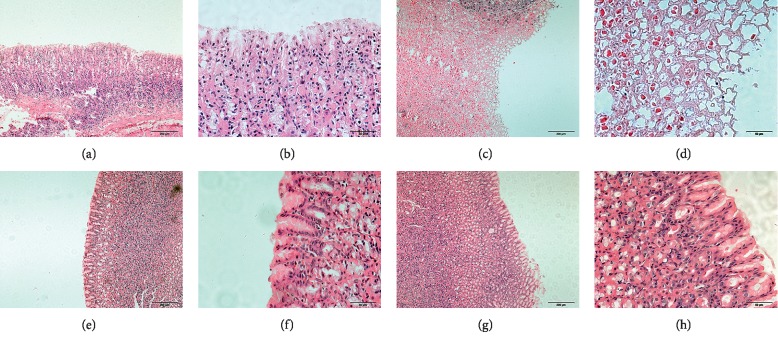
Pathological morphology examination of gastric mucosa in rats of each group. (a, b), mean controls; (c, d), mean AGU groups; (e, f), mean ST group; and (g, h), mean GB group. The scale bars in a c, e, and g represent 200 *μ*m, and the scale bars in b, d, f, and h represent 50 *μ*m.

In the control group, the gastric mucosa cells were arranged orderly, the gastric mucosa was intact, and there were no mucosal damage or exfoliation. On the contrary, the gastric mucosa in the AGU group was damaged obviously, and the cells arranged in disorder. Compared with the AGU group, the surface of gastric mucosa in the ST group was partially shedding, the cells arranged orderly, and new mucosal layers were observed. In the GB group, the gastric mucosa was still damaged in a small part. The arrangement of gastric mucosa cells was more orderly than that in the model group, but the normal arrangement of gastric mucosa cells had not yet been restored (Figure 2).

### 3.3. Quantitative Real-Time PCR (qPCR) Assessment

**Figure 3:**
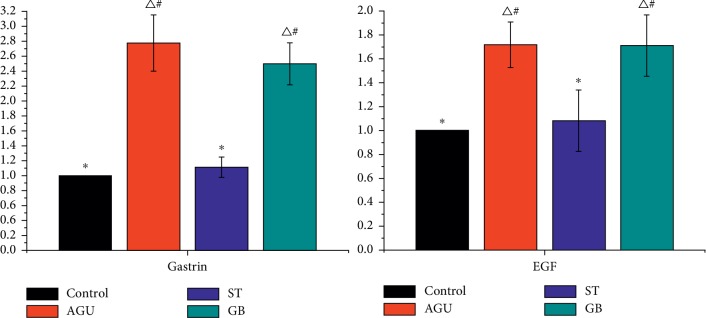
The expression of GAS and EGF in gastric mucosa in rats of each group (^Δ^significant difference from the control group at *P* < 0.05, ^*∗*^significant difference from the AGU group at *P* < 0.05, ^#^means difference from the ST group at *P* > 0.05).

The relative expressions of GAS and EGF in gastric mucosa of AGU rats were detected by qPCR. The relative expression of GAS and EGF in gastric mucosa of AGU and GB rats showed higher expression than that in control. On the contrary, there was no significant difference in the relative content of GAS and EGF between the ST group and control group (Figure 3).

### 3.4. Relative Content of Bacteria

**Figure 4:**
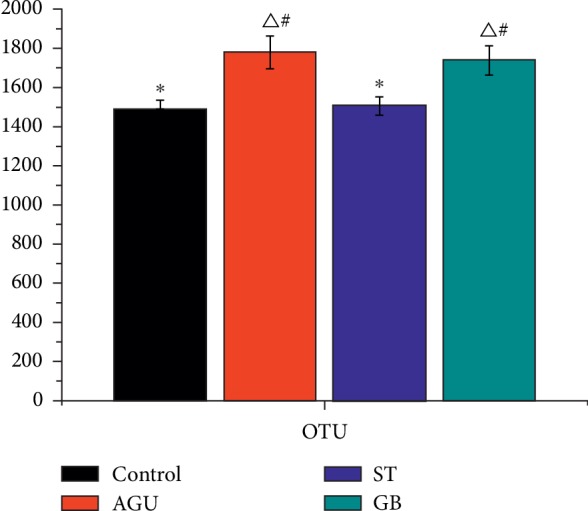
OUT abundance of feces in each group (^Δ^significant difference from the control group at *P* < 0.05, ^*∗*^significant difference from the AGU group at *P* < 0.05, ^#^means difference from the ST group at *P* > 0.05).

In this study, the dual terminal data were obtained from the Illumina Hiseq sequencing platform. Then, the OTU abundance of each sample was analyzed by QIIME software for OTU clustering. The OTU abundance in the control group was lower than that in the AGU group significantly. The OTU abundance in the ST group was lower than that in the GU group significantly. However, the OTU abundance in the GB group was not different significantly from the GU group (Figure 4).

### 3.5. Characterization of Microbiota

**Figure 5:**
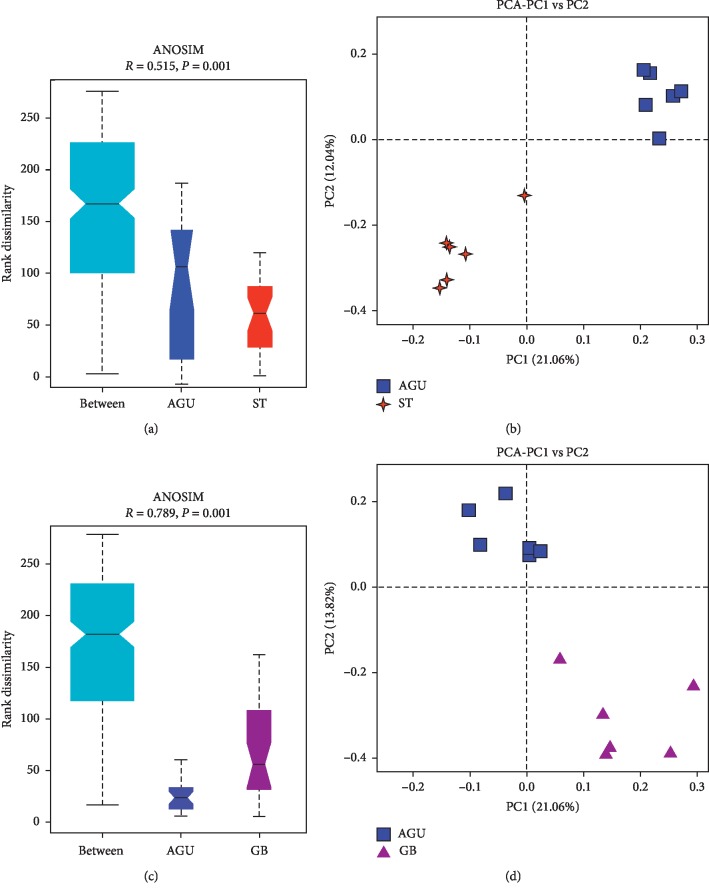
Analysis of similarities (ANOSIM) and PCA analysis in (a, b) the AGU group and ST group and (c, d) the AGU group and GB group.

PCA analysis and analysis of similarities (ANOSIM) were used to statistics the microbiome of rats in each group, indicating that the microbiome in each group was significantly different (Supplementary [Supplementary-material supplementary-material-1]). On the basis of the results of PCA analysis in the AGU group and the ST group, it was found that the two groups had better dispersion, and the two groups had significant differences. The results of the PCA were showing that the AGU group and the GB group were comparable. At the same time, the results also showed the bacteria of the AGU group and the GB group had good dispersion, and the two groups of bacteria had significant differences (Figure 5).

### 3.6. Species Changes of Intestinal Microbiome

**Figure 6:**
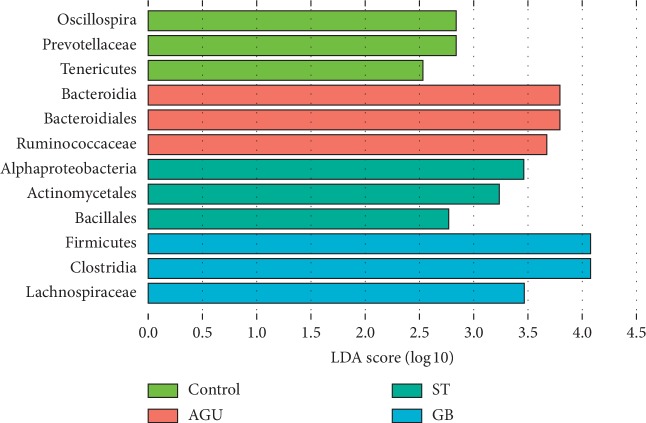
The histogram of LDA value distribution in four groups. Three species with the most difference significantly were selected in four groups.

To achieve the comparison flora among the groups, different abundance between the groups of the species were analyzed through LEfSe. Meanwhile, a comparative analysis of the subgroups among the four groups could be used to obtain a high relative population between the groups. The results of the three most diverse and highest expressions in each group were selected in experiment. According to the results, the dominant species of the control group were Oscillospira, Prevotellaceae, and Tenericutes. Then, the dominant species of the AGU group were Bacteroides, Bacteroidales, and Ruminococcaceae. At the same time, Alpha-proteobacteria, Actinomycetales, and Bacillales were the dominant species in the ST group, while, Firmicutes, Clostridia, and Lachnospiraceae were dominant species in the GB group (Figure 6). According to the ratio of *F*/*B*, there was significant difference in the ratio between the ST group and AGU group. However, there was no significant difference between the ST group and control group (Supplementary [Supplementary-material supplementary-material-1]).

## 4. Discussion

The clinical manifestations of gastric ulcer were stomachache, anorexia, belching, and so on [21, 22]. The ulcer index and water intake of gastric ulcer rats could reflect the degree of gastric mucosa lesions in rats. According to the abovementioned research data, illustrating that moxibustion on the acupoints of stomach meridian could repair the gastric mucosal lesions. Oppositely, moxibustion on the acupoints of the gallbladder meridian had little effect on the repair of gastric mucosa.

Brain-gut peptide (BGPs) is the main factor in repairing gastric mucosal injury. BGPs not only regulate activity of the central nervous systems, but also play a vital role in gastrointestinal motility [23–25]. GAS and EGF are the main substances of brain-gut peptide. Gastrin (GAS) is mainly mediated by the gas receptor to stimulate the growth of gastric mucosal cells and maintains the integrity of the mucosa [24]. However, EGF mainly increases gastric mucosal blood flow and inhibits apoptosis of gastric mucosal cells, thus maintaining gastrointestinal mucosal integrity [26, 27]. In this research, both substances were reversed to normal levels after intervention by moxibustion, which means moxibustion achieved therapeutic effects by regulating brain-gut peptides.

The intestinal microbiome is rich in the human body. These species of bacteria purposely stabilize the digestive function of human [28]. The recent research shows that the flora in the digestive tract can be divided into dominant flora and inferior flora. The dominant flora indicates the large quantity in the intestine, which is most related with digestive functions [29]. In the other words, the inferior flora is expressed with less quantity in the gastrointestinal tract, which is a group that is less related to the digestive functions. However, when the gastrointestinal tract pathologically changes, the dominant flora may transform into inferior flora, and the inferior flora may transform into dominant flora too [30]. Therefore, the relationship between the functional status of the digestive tract and dominant flora is very important. In this project, moxibustion treatment can reverse the different levels in the intestinal flora of the fecal samples caused by AGU modeling. These results are discussed in detail below. In addition, the intestinal environment may be changed by increasing the Firmicutes/Bacteroidetes ratio and then can improve immune function and promote bowel health [31]. According to the research results, it was found that the ST group can significantly increase the ratio of F/B, indicating that the ST group had a good effect on the increase of intestinal beneficial bacteria.

In this research, the three highest relative expression dominant groups in the control group were Oscillospira, Prevotellaceae, and Tenericutes. Oscillospira is the dominant flora which exists in the normal human body [32]. It can maintain the immune role of the digestive tract, and it has an anti-inflammatory effect when there is an inflammatory reaction in the digestive tract [33]. Prevotellaceae is one of the preponderant bacteria in the human intestinal tract. It not only maintains the balance of intestinal flora but also plays an important role in maintaining the normal physiological function of the digestive tract. Studies have shown that [32] Prevotellaceae can effectively regulate the intestinal flora after diarrhea and return to normal under intestinal flora balance [34]. Tenericutes is a good group of flora in the intestine. It maintains the normal state of the intestinal immune system [35]. While the quantity of Tenericutes reduces, it will cause inflammation and lead to intestinal immune dysfunction [36]. Therefore, these first three dominant bacteria groups are beneficial bacteria in the normal specimen intestinal tract. They are important to maintain the normal physiological state of the intestinal tract.

The three highest relative expression dominant groups in the AGU were Bacteroidia, Bacteroidales, and Ruminococcaceae. Bacteroides does not cause pathogenic effects in normal conditions. However, it can become a pathogen under certain pathological conditions [37]. Meanwhile, Bacteroides is abundant in normal humans and animals. In some animal specimen of inflammatory bowel disease, Bacteroides becomes pathogenic bacteria, which show certain inflammatory response characteristics. Early studies found that the quantity of Bacteroides in DS specimen increases, which means that the increase in Bacteroides may involve in the pathogenesis of intestinal inflammation in the specimen [38]. Bacteroides is a pathogenic bacterium. When it becomes a dominant bacterium, it can promote intestinal inflammation in the intestine. The substance secreted by it has membrane aggressiveness, which can destroy the mucosal morphology and injured the mucosa of the gastrointestinal tract [39]. The Ruminococcaceae is a beneficial bacteria in the digestive tract [40]. When there is inflammation in the alimentary canal, it can proliferate to promote the repair of the alimentary canal inflammation. Therefore, the AGU specimen shows that the pathogenic bacteria will become dominant species and can destroy the gastrointestinal mucosa. However, due to the self-repair function of specimen themselves, there are still beneficial bacteria in the inflammatory specimen to inhibit the production of pathogenic bacteria, which is an indication of the function of self-healing in specimen with AGU [41].

In the ST group, the three highest relative expression dominant microflora were Alphaproteobacteria, Actinomycetales, and Bacillales. It is an important beneficial self-defence bacterium in the body. Moxibustion can effectively mobilize the intestinal Alphaproteobacteria in the acute gastric ulcer (AGU) specimen for self-inflammatory immune defense. There should be an important regulatory relationship between Zusanli (ST36), Liangmen (ST21), and Alphaproteobacteria [42]. Actinomycetales is the dominant species in the intestine [43]. It has antitumor functions and inhibits the activity of pathogenic bacteria. It also protects the species and quantity of intestinal flora [44]. The quantity of Actinomycetales increases indicating that moxibuction is effective. The treatment of gastrointestinal diseases through moxibustion Zusanli (ST36) and Liangmen (ST21) should have an important relationship with the regulation of actinomycetes. Bacillales is an intestinal probiotic that plays an important role in promoting intestinal peristalsis [45], indicating that moxibustion Zusanli (ST 36) and Liangmen (ST21) can promote gastrointestinal peristalsis by increasing the relative abundance of Bacillales, which is of great significance for exploring the mechanism of action of gastric meridians and gastrointestinal motility.

The three highest relative expression dominant groups in the GB group were Firmicutes, Clostridia, and Lachnospiraceae. Several studies have shown that Firmicutes have an important role in energy absorption which can absorb energy in food more effectively, so it has an important correlation with changes in body weight [46]. The energy consumption of the body will increase when the inflammation of the digestive tract is produced. Therefore, to eliminate inflammation in the digestive tract, the better energy assorted is by increasing the abundance of the Firmicutes. Clostridium is a pathogenic bacterium that will lead to intestinal inflammation when it exists in the intestinal tract [47]. The specimen in this AGU experiment showed that this pathogenic bacterium will affect the normal function and lead to intestinal dysfunction of the intestinal tract. Lachnospiraceae is a beneficial bacterium in protecting the gastrointestinal mucosa and preventing mucosal carcinogenesis which is often used in the study of anticolon cancer [48]. Thus, it has the reflection of self-immune and self-healing in the specimen by increasing the abundance of Lachnospiraceae [48]. Therefore, when moxibustion is used to treat acute gastric ulcer (AGU) on nonacupoints), there are still pathogenic bacteria in the intestinal tract, and the relative abundance of pathogenic bacteria is higher than the effect of beneficial bacteria.

Together, the dominant bacteria belonging to the control group purposely have an immune role in the intestinal tract and maintain the balance of intestinal flora in human body. The dominant bacteria belong to the AGU group were mainly pathogenic bacteria, which showed proinflammatory response, in different damage levels to the gastric mucosa. The dominant bacteria belonging to the ST group were mainly eliminating the intestinal inflammatory reaction and maintain the relative stability of the gastrointestinal environment. The dominant bacteria in the GB group showed that the pathogenic bacteria coexisted with the beneficial bacteria, and the intestinal microenvironment is between the GB group and the ST group.

In conclusion, moxibustion acupoints had significant effect on AGU. It could repair the pathological damage of gastric mucosa in specimens with AGU and reduce the area of ulcer effectively. At the same time, it could regulate the imbalance of GAS and EGF in gastric mucosa with acute gastric ulcer to promote the repair of gastric mucosal injury. The most important was that moxibustion can also improve the intestinal flora imbalance in the specimen with AGU by regulating the quantity and species of intestinal flora, which was also increasing the quantity and species of intestinal beneficial bacteria. The observation of changes in intestinal microbiome by macrogene sequencing will further clarify the mechanism of action of moxibustion acupoints on AGU. The current work will help to better understand the differential changes in intestinal microbiome during the treatment of AGU with moxibustion.

## Figures and Tables

**Table 1:** Gastric ulcer index in all groups.

Groups	Ulcer index
Control	0.00 ± 0.00^*∗*^
AGU	6.83 ± 1.33^Δ#^
ST	2.50 ± 1.22^*∗*^
GB	6.67 ± 0.81^Δ#^

Data are presented as mean ± standard error of the mean. ^Δ^significant difference from the control group at *P* < 0.05, ^*∗*^significant difference from the AGU group at *P* < 0.05, ^#^means difference from the ST group at *P* > 0.05.

## Data Availability

The original data used to support the findings of this study were supplied by Zongbao Yang under license and so cannot be made freely available. Requests for access to these data should be made to Zongbao Yang, yangzb@xmu.edu.cn.

## Supplementary Materials

Supplementary Materials Supplementary Figure S1: the location of acupoints for Zusanli (ST 36, located 5 mm below the fibular head and lateral to the anterior tubercle of the tibia) and Yanglingquan (GB 34, located 5 mm above the outer side of ST 36) on rats (a); the location of acupoints for Liangmen (ST 21, located 5 mm horizontally to the spot above 2 cm from the navel) and Riyue (GB 24, located on the middle line of clavicle, the lower edge of the seventh rib) on rats (b); and moxa cones (c). Supplementary Figure S2: analysis of similarities (ANOSIM) and PCA analysis between the control group and AGU group (*A*); analysis of similarities (ANOSIM) and PCA analysis between the ST group and GB group (B); and analysis of similarities (ANOSIM) and PCA analysis among all the groups (C). Supplementary Figure S3: the ratio of Firmicutes to Bacterioidetes in fecal flora of rats in each group (? significant difference from the control group at *P* < 0.05, ^*∗*^significant difference from the AGU group at *P* < 0.05, and ^#^means difference from the ST group at *P* > 0.05).
