# Supplementary material for: Effect of Moxibustion on Intestinal Microbiome in Acute Gastric Ulcer Rats
Source: Evid Based Complement Alternat Med. 2019 Dec 18;2019:6184205. doi: 10.1155/2019/6184205 (PMC6948313; doi:10.1155/2019/6184205)
Supplement: Supplementary Materials — Supplementary Figure S1: the location of acupoints for Zusanli (ST 36, located 5 mm below the fibular head and lateral to the anterior tubercle of the tibia) and Yanglingquan (GB 34, located 5 mm above the outer side of ST 36) on rats (a); the location of acupoints for Liangmen (ST 21, located 5 mm horizontally to the spot above 2 cm from the navel) and Riyue (GB 24, located on the middle line of clavicle, the lower edge of the seventh rib) on rats (b); and moxa cones (c). Supplementary Figure S2: analysis of similarities (ANOSIM) and PCA analysis between the control group and AGU group (A); analysis of similarities (ANOSIM) and PCA analysis between the ST group and GB group (B); and analysis of similarities (ANOSIM) and PCA analysis among all the groups (C). Supplementary Figure S3: the ratio of Firmicutes to Bacterioidetes in fecal flora of rats in each group (? significant difference from the control group at P < 0.05, ∗significant difference from the AGU group at P < 0.05, and #means difference from the ST group at P > 0.05). [file 6184205.f1.pdf]

Effect of moxibustion on intestinal microbiome in acute gastric ulcer rats

Qi-da He[1,3], Miao-sen Huang†[1,3], Long-bin Zhang[1], Jia-cheng Shen[1], Lin-yu Lian[1,3],Yuan Zhang[1,2], Bao-hua Chen[1], Cai-chun Liu[1], Lin-chao Qian[1], Mi Liu[2], Zong-bao Yang[1※]

[1] Cancer Research Center, School of Medicine, Xiamen University, Xiamen 361005, China

[2] College of Acupuncture and Moxibustion, Hunan university of Traditional Chinese Medicine, Changsha 410208, China

[3] College of Acupuncture and Moxibustion, Fujian university of Traditional Chinese Medicine, Fuzhou 350122, China

† Miao-sen Huang is the co-first author.

※ Correspondence should be addressed to Zong-bao Yang, yangzb@xmu.edu.cn and Mi-Liu, newmean9722@qq.com.

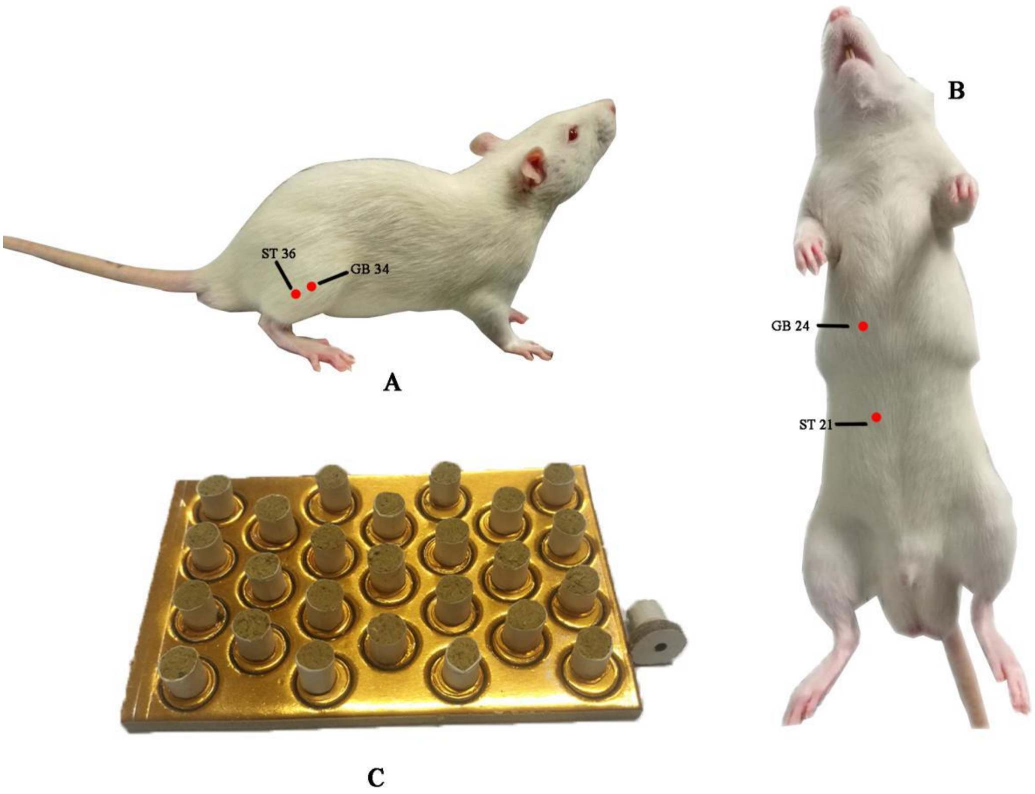

Figure S1: The location of acupoints for Zusanli(ST 36, located 5 mm below the fibular head and lateral to the anterior tubercle of the tibia) and Yanglingquan(GB 34,located 5mm above the outer side of ST 36) on rats (a); The location of acupoints for Liangmen(ST 21, located 5 mm horizontally to the spot above 2cm from navel) and Riyue(GB 24,located on the middle line of clavicle, the lower edge of the seventh rib) on rats(b); moxa cones(c).

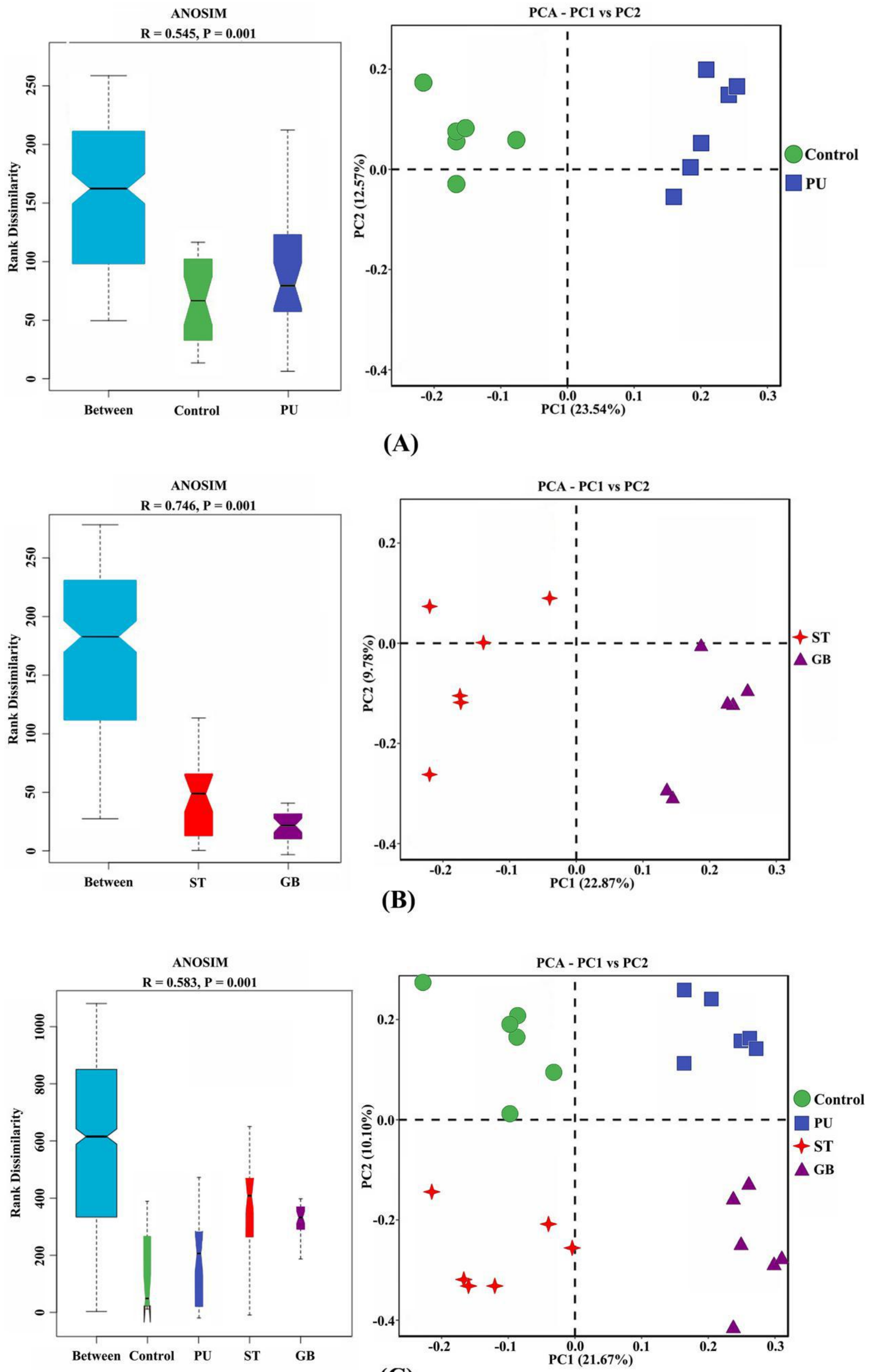
ANOSIM
R = 0.545, P = 0.001
Rank Dissimilarity
0
50
100
150
200
250
Between
Control
PU
PCA - PC1 vs PC2
PC2 (12.57%)
PC1 (23.54%)
0.2
0.0
-0.2
-0.4
-0.2
-0.1
0.0
0.1
0.2
0.3
Control
PU
(A)
ANOSIM
R = 0.746, P = 0.001
Rank Dissimilarity
Between
ST
GB
PCA - PC1 vs PC2
PC2 (9.78%)
PC1 (22.87%)
ST
GB
(B)
ANOSIM
R = 0.583, P = 0.001
Rank Dissimilarity
0
200
400
600
800
1000
Between
Control
PU
ST
GB
PCA - PC1 vs PC2
PC2 (10.10%)
PC1 (21.67%)
Control
PU
ST
GB
(C)

Figure S2: Analysis of similarities (ANOSIM) and PCA analysis between control group and AGU group (A) ; Analysis of similarities (ANOSIM) and PCA analysis between ST group and GB group (B) ; Analysis of similarities (ANOSIM) and PCA analysis among all the groups (C).

PCA analysis and analysis of similarities (ANOSIM) were used to statistics the microbiome of rats in each group. The result indicated that the control group and the AGU group were comparable. On the basis of the results of PCA analysis in the control group and AGU group, which was found that the two groups had significant differences. The results suggested that the ST group and the GB group were comparable. At the same time, the results also showed the two groups of bacteria had good dispersion, and the two groups of bacteria had significant differences. Meanwhile, according to the PCA and ANOSIM analysis results could be known that all groups of bacteria have significant differences. (**Figure S2**)

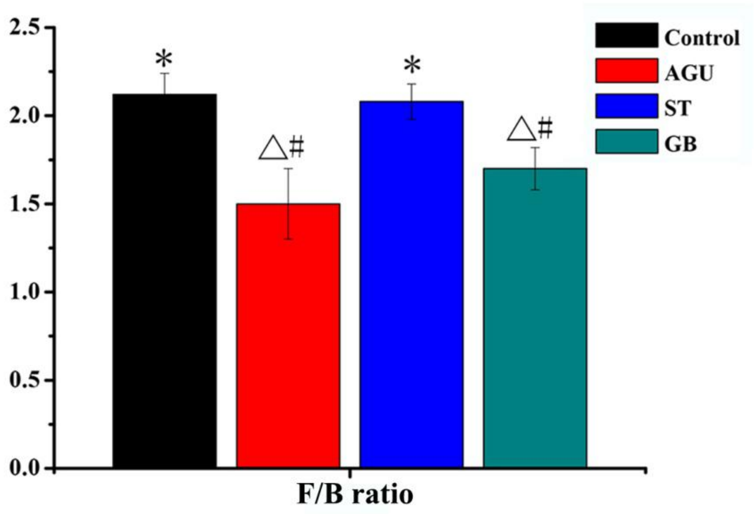

Figure S3: The ratio of Firmicutes to Bacterioidetes in fecal flora of rats in each group. (△significant difference from the control group at $P < 0.05$, * significant difference from the AGU group at $P < 0.05$, #means difference from the ST group at $P > 0.05$.)
